# A Remarkable Presentation of Phyllodes Tumor With Pseudoangiomatous Stromal Hyperplasia

**DOI:** 10.7759/cureus.71900

**Published:** 2024-10-20

**Authors:** Arunima Das, Madan Sundar, Magesh Chandran, Kuberan Krishnan

**Affiliations:** 1 General Surgery, Sree Balaji Medical College and Hospital, Chennai, IND; 2 Surgery, Bharath Institute of Higher Education & Research, Chennai, IND; 3 General Surgery, Bharath Institute of Higher Education & Research, Chennai, IND

**Keywords:** clinical outcome, cystosarcoma phyllodes, fibroadenoma-like changes, fibroepithelial tumor, immunohistochemistry(ihc), pathological features, pseudoangiomatous stromal hyperplasia (pash), stromal changes

## Abstract

Phyllodes tumor is a rare and particular type of breast tumor with features of stromal hyperplasia and malignant potential. The present case is an unusual presentation of a phyllodes tumor with pseudoangiomatous stromal hyperplasia (PASH) which forms an essential part of benign breast disease but is not frequently described in association with phyllodes tumor. A 36-year-old female patient was referred to the hospital with a large breast mass considered to be a benign growth. Histopathological examination revealed it to be a phyllodes tumor with a feature of pseudoangiomatous stromal hyperplasia. This case report contributes to the understanding of the phyllodes tumor and emphasizes differential diagnosis in breast pathology. It shows the need for histopathological assessment in order to distinguish between these tumors and to determine their treatment.

## Introduction

Phyllodes tumors or cystosarcoma phyllodes are breast tumors that are of fibroepithelial histogenesis and may be benign or malignant. These tumors are seen to have a biphasic pattern containing both stromal and epithelial components [1], but the stroma is the dominant component of the tumor and therefore determines the growth pattern. They feature a stromal component with a leaf-like architecture and varying degrees of cellularity. They may be classified as benign, borderline, or malignant based on the extent of stromal overgrowth, nuclear atypia, and mitotic activity. Although they are not very common, occurring in less than 1% of all breast tumors, phyllodes tumors still present a diagnostic and therapeutic challenge due to their variable aggressiveness and high propensity for local relapse and metastases. Pseudoangiomatous stromal hyperplasia (PASH) is a benign breast disease characterized by the increase of stromal cells that resemble blood vessels but are not true vessels [2]. It clinically mimics fibroadenoma or benign phyllodes tumor, and histologically it is similar to low-grade angiosarcoma. Although common in benign breast diseases like fibrocystic changes or fibromas such as PASH, it is rarely reported with breast carcinoma either naturally accompanying or associated in the same or contralateral breast [3-4]. The association of PASH with phyllodes tumor poses a diagnostic challenge that requires a clear understanding of the two conditions as a way of preventing the mismanagement of way of patients with this condition.

## Case presentation

A 36-year-old multiparous female patient came to the surgery outpatient department as she noticed a lump in her right breast for the last 4 years and she also noticed another lump in her left breast 2 months back. Both the lump was painless and nonprogressive in nature. On examination: An ovoid-shaped lump with a size of 3x4 cm was palpable in the lower outer quadrant of the right breast which was firm in consistency and mobile, and the skin over the lump was pinchable. An ovoid-shaped lump with a size of 3x3 cm was palpable in the lower outer quadrant of the left breast which was firm in consistency, mobile, and with skin over the lump pinchable.

**Figure 1:**
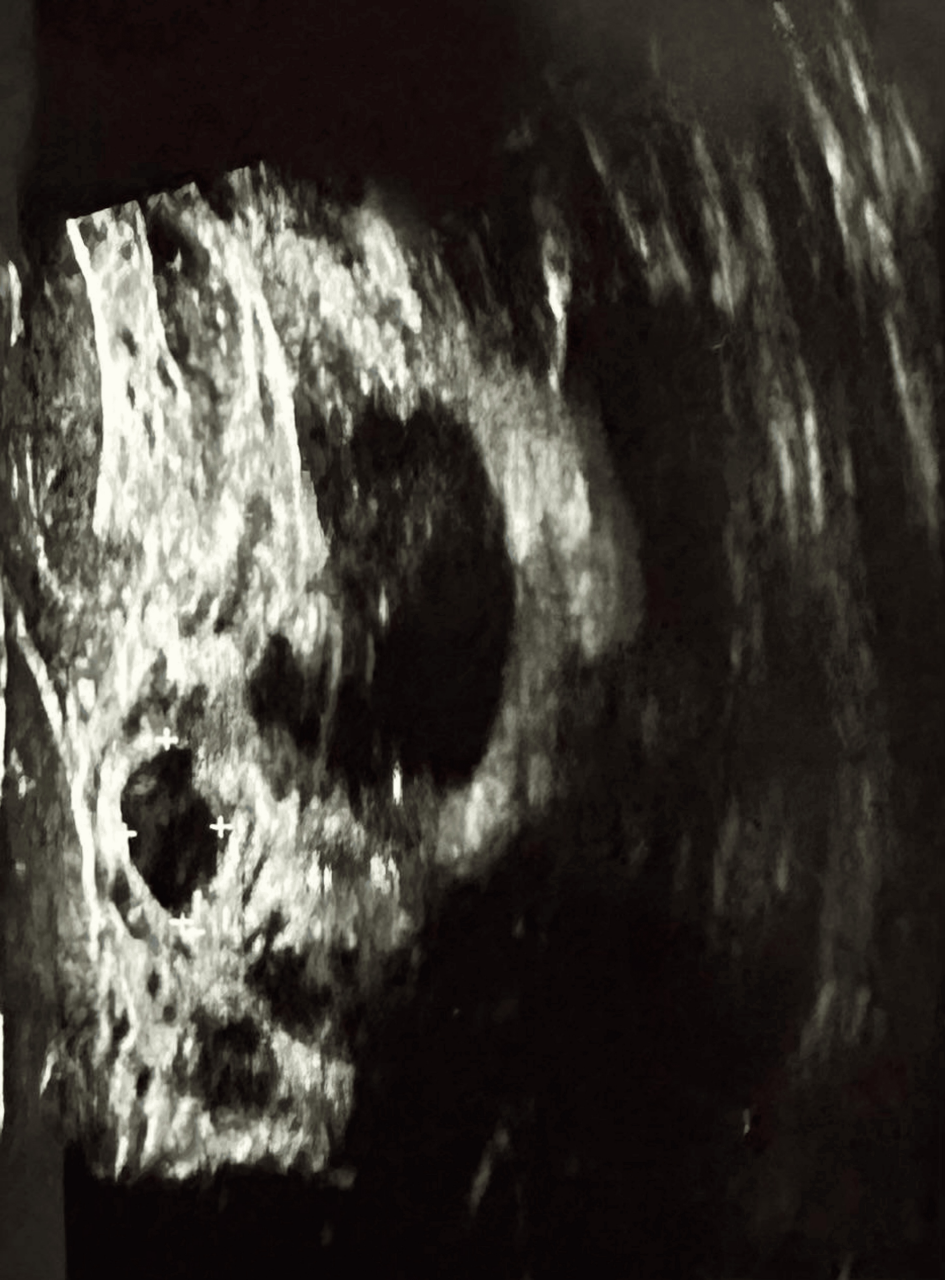
Ultrasonography findings: hypoechoic lesion of the right breast

**Figure 2:**
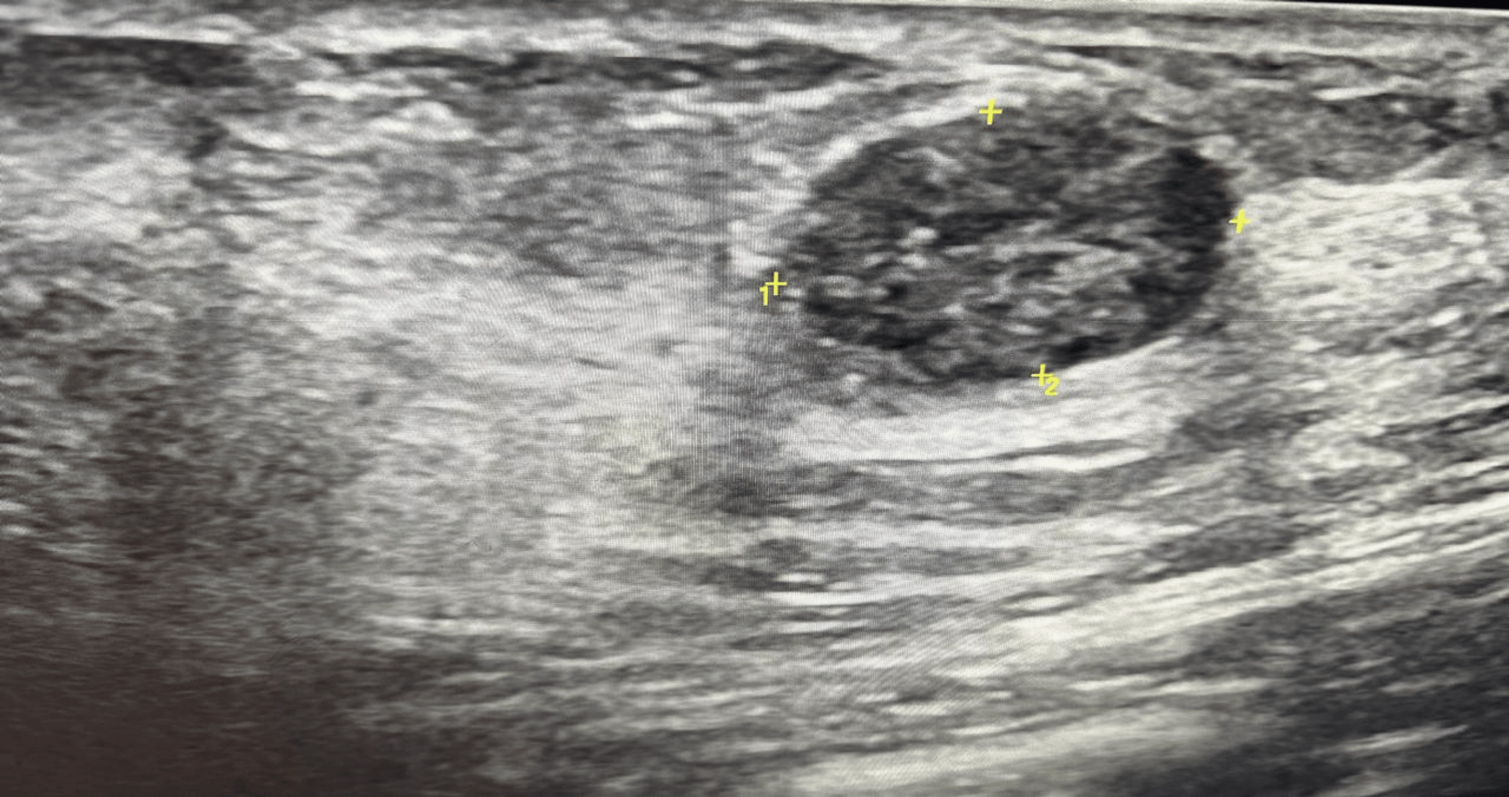
Ultrasonography findings of left breast: hypoechoic lesions with some calcification

Ultrasonography (USG) bilateral breast showed multiple well-defined oval hypoechoic lesions of size 2.8x1.4 cm in the right breast (Fig. 1) and 3.1 x 1.6 cm in the left breast (Fig. 2) with calcification and shadowing seen in both breasts which was suggestive of fibroadenomas with calcification (BI-RADS (Breast Imaging-Reporting and Data System) III/IVA).

Tru-cut biopsy was done for bilateral breast and it showed fibroadenoma with sclerosed stroma (negative for malignancy) for both breasts. The patient was planned for an excisional biopsy of bilateral breast swellings.

**Figure 3:**
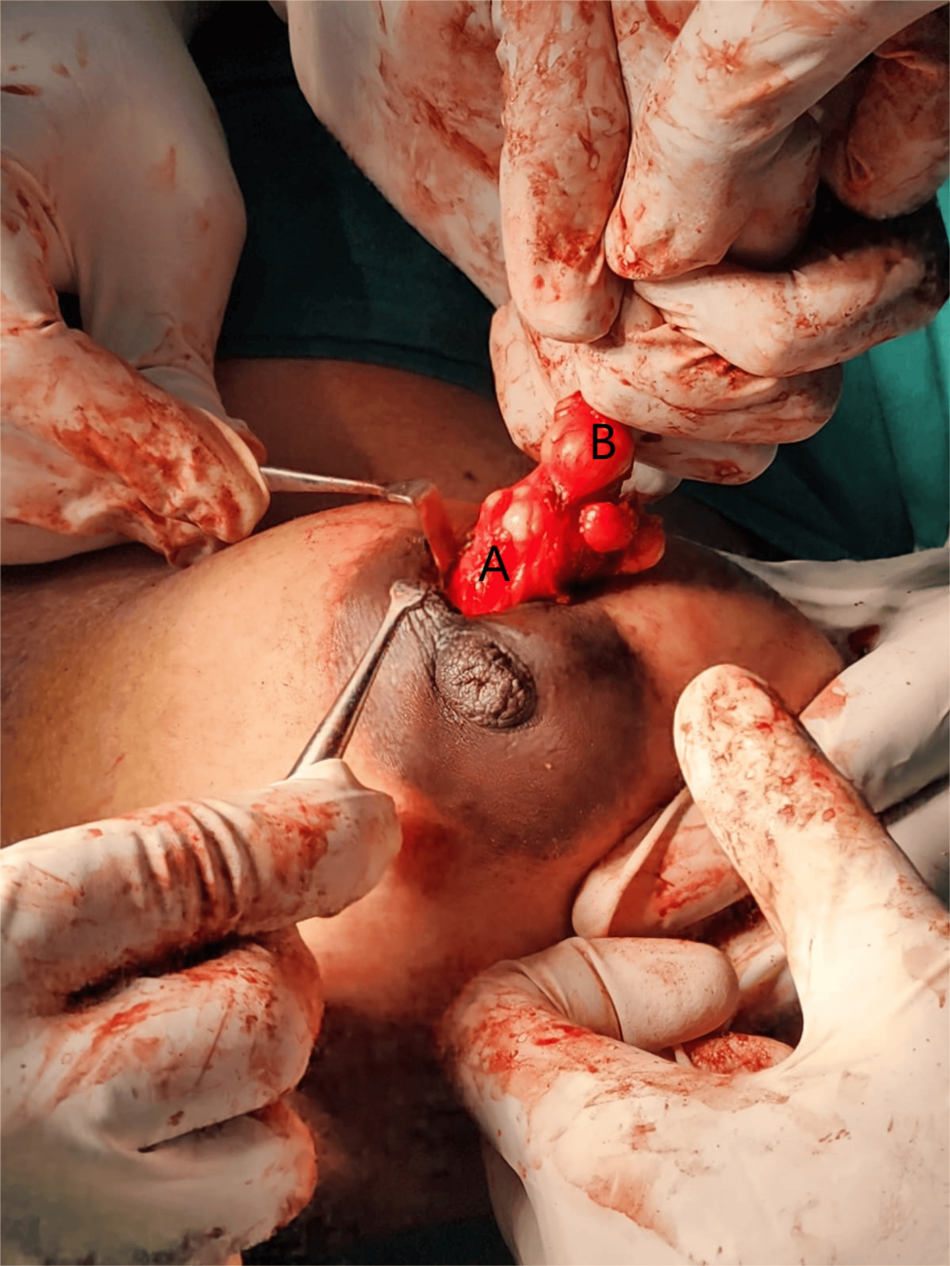
Excision and biopsy of the right breast: A - fibroepithelial tumour (phyllodes tumor); B - fibrocystic disease with pseudoangiomatous stromal hyperplasia (PASH)

**Figure 4:**
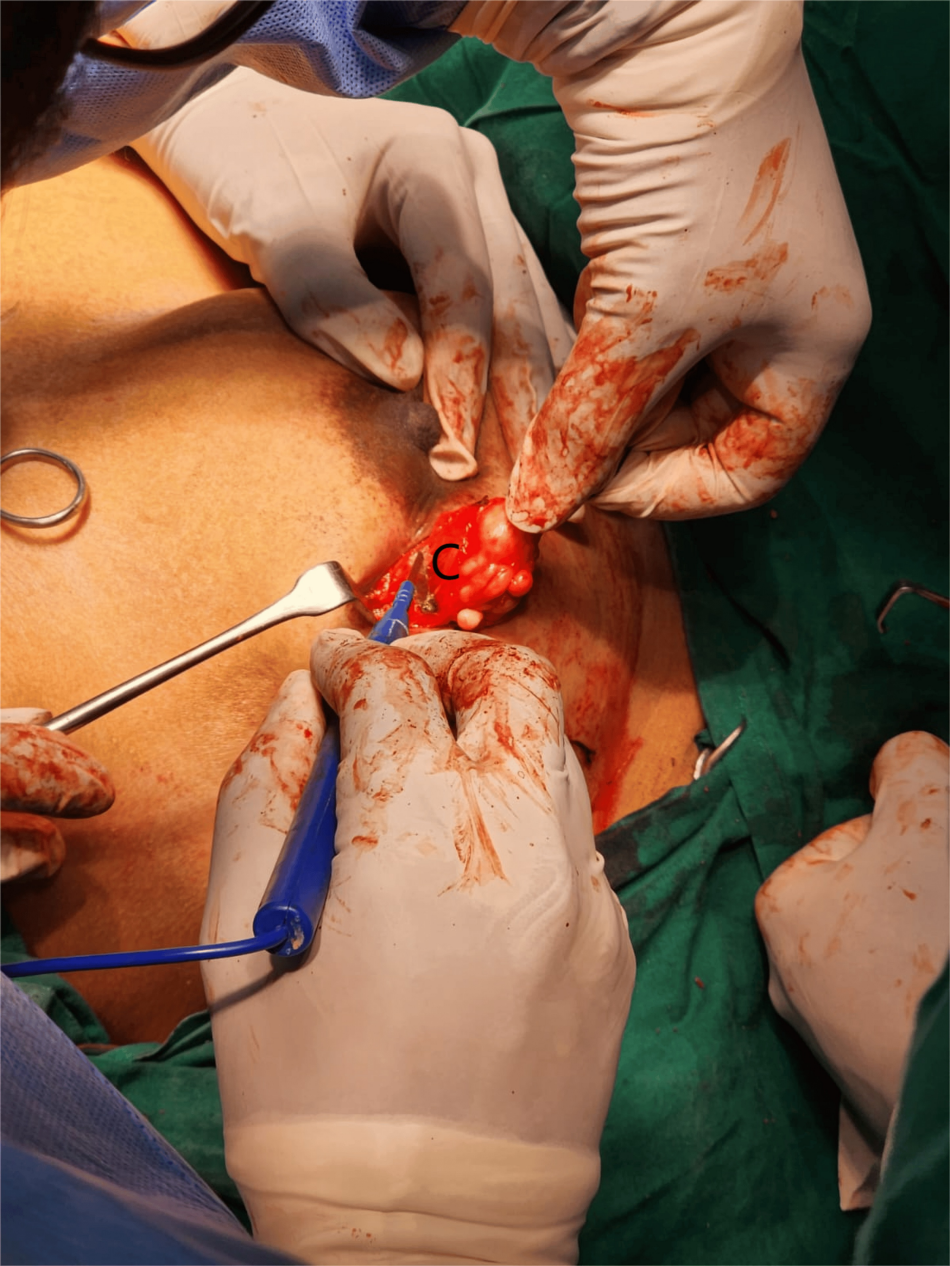
Excision and biopsy of the left breast: C - fibroadenoma with degenerative changes and dystrophic calcification

A circumareolar incision was made and deepened over the right breast. A lump of size 4x3 cm was excised in toto with a 1 cm margin (Fig. 3). In the cut section, variable consistency was noted - putty with firm consistency noted along with debris. A circumareolar incision was made and deepened over the left breast. A lump of size 4x3 cm was excised in toto which was soft in consistency (Fig. 4). 

**Figure 5:**
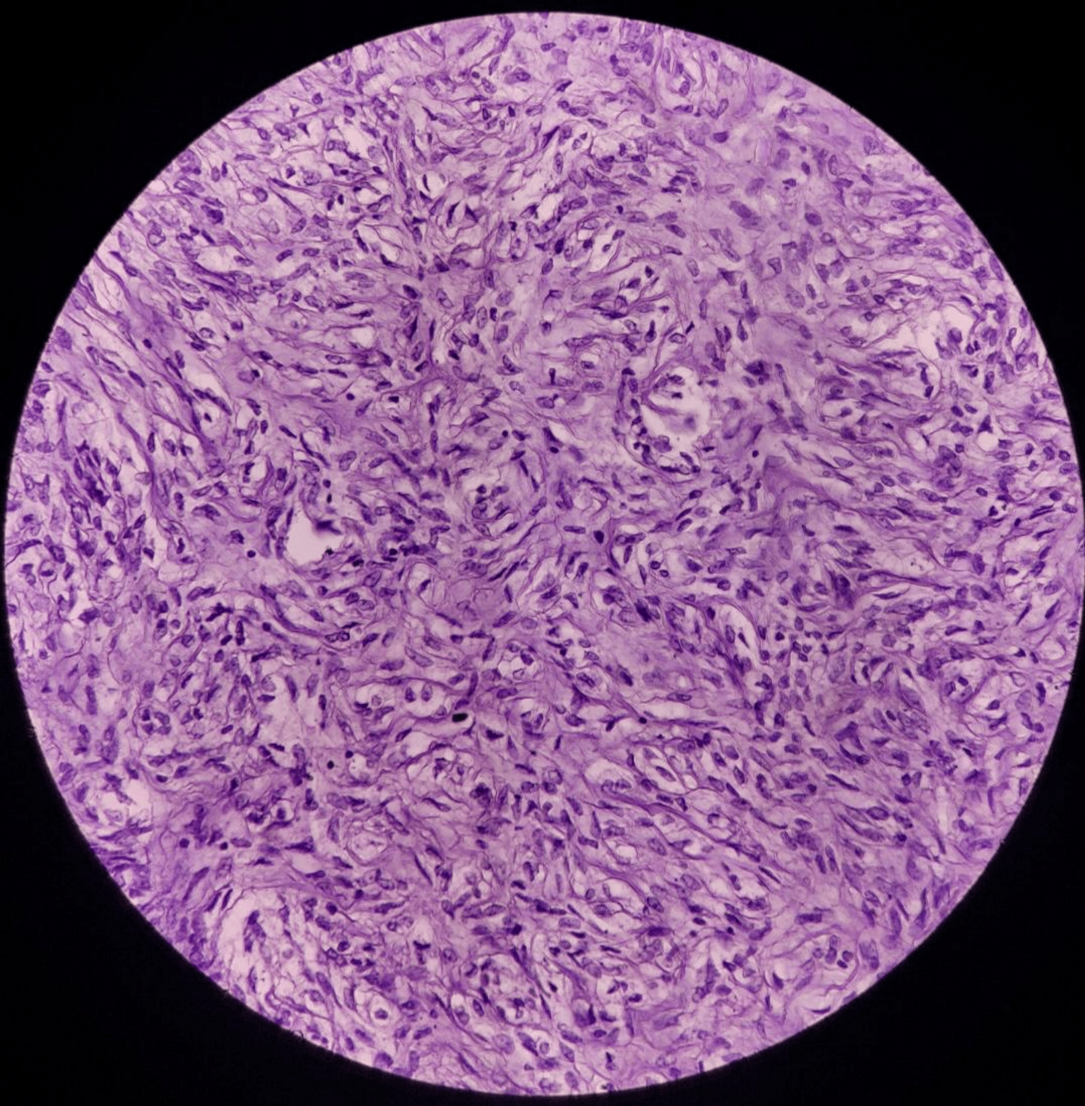
Fibroepithelial tumor with extensive sclerosis of stroma (phyllodes tumor) suggestive of P63

**Figure 6:**
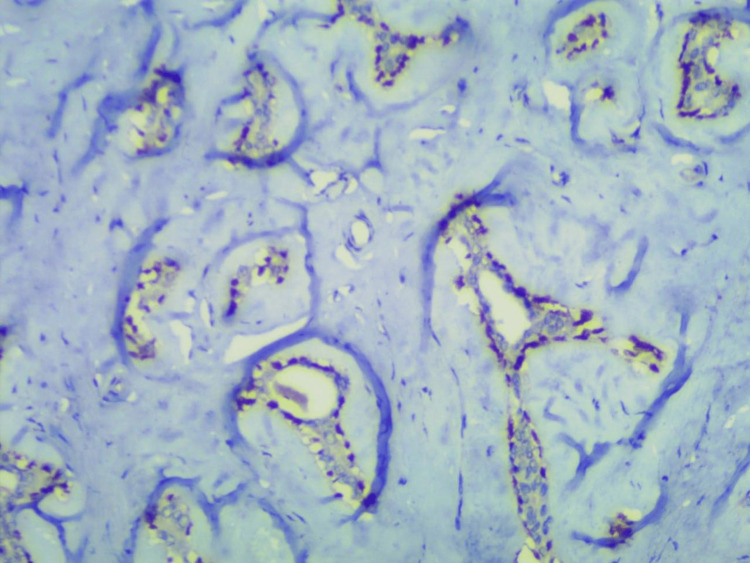
Immunohistochemistry P63: positive in myoepithelial cells

**Figure 7:**
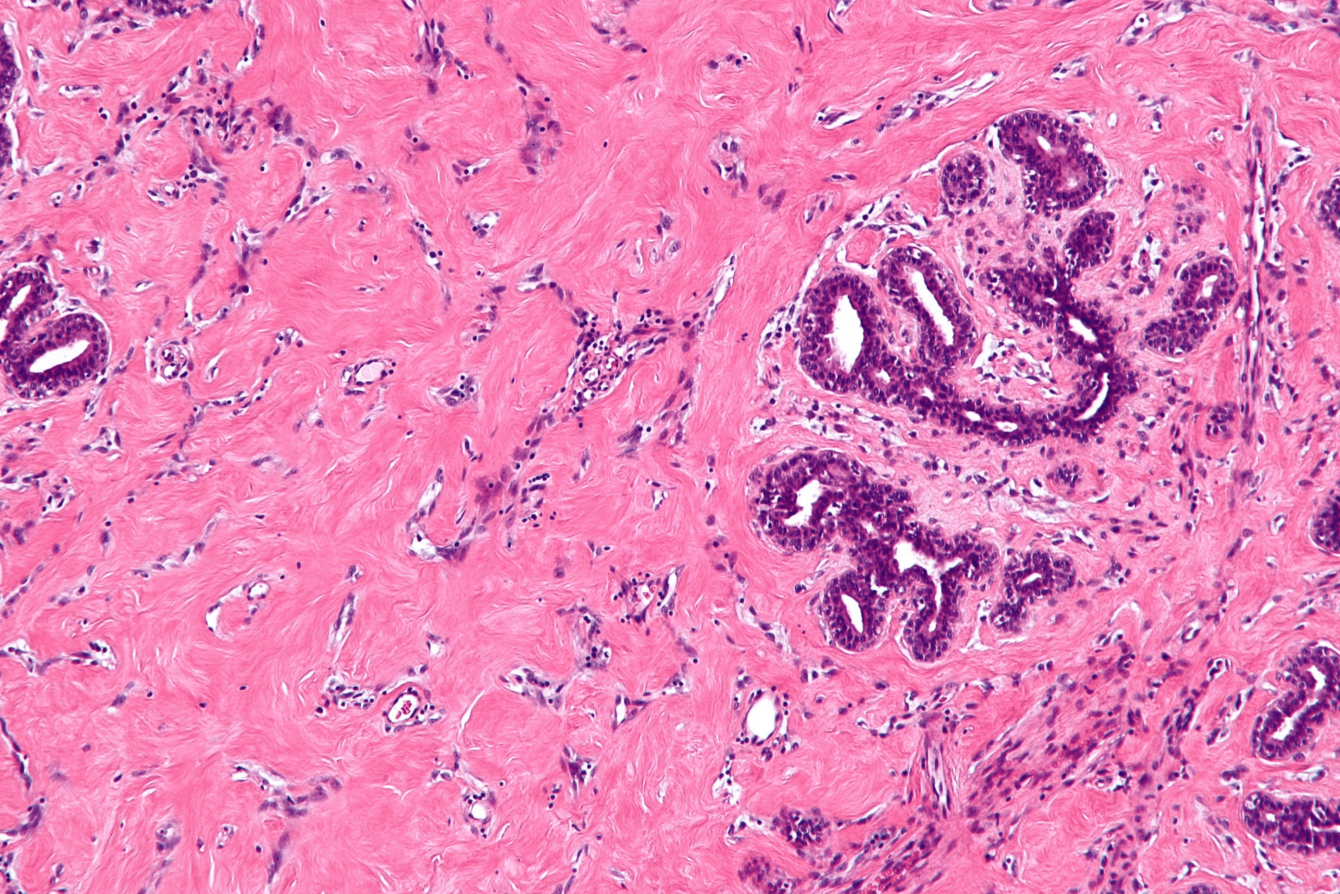
Fibrocystic disease with pseudoangiomatous stromal hyperplasia (PASH)

Histopathological examination of the right breast lump showed a fibroepithelial tumor with extensive sclerosis of the stroma, however, the epithelial component showed hyperplasia with an opened-up nucleus and conspicuous nucleoli (Fig. 5), which required P63 staining by immunohistochemistry, positive in myoepithelial cells denotes benign nature of the tumor (Fig. 6). A section of tissue showed features of fibrocystic disease and pseudoangiomatous stromal hyperplasia (Fig. 7). The left breast lump histopathology showed features of fibroadenoma.

After surgical excision, the patient was on follow-up for 6 months. There was no evidence of any complications or recurrence.

## Discussion

Pseudoangiomatous stromal hyperplasia (PASH) is most frequently observed in women of reproductive age, particularly those who are premenopausal or undergoing hormone replacement therapy. Hormonal influences, particularly from progesterone, are thought to play a significant role in the development of PASH, which can be aggravated by hormonal fluctuations [5]. This condition can also present in adolescents and pregnant women [6-8].

Both PASH and fibroadenomas typically manifest as well-circumscribed, palpable masses [9]. While they are often painless, PASH can sometimes be painful or exhibit rapid growth. Fibroadenomas are usually detected during routine breast examinations or imaging studies, whereas PASH may go unnoticed until discovered incidentally on imaging performed for another reason, as it can sometimes be asymptomatic or less prominent than fibroadenomas.

Tumoral or nodular PASH is best identified through ultrasound, where its characteristics are assessed based on shape, circumscription, margins, echogenicity, vascularity, and posterior acoustic features as per BI-RADS guidelines. Tumoral PASH typically appears as a well-circumscribed, oval, or round mass parallel to the chest wall, commonly exhibiting homogeneous hypoechogenicity. Occasionally, it may present with complex echogenicity and irregular shapes. Rare cases may show ill-defined margins, making it difficult to differentiate from malignancy. Small internal hypoechoic or anechoic areas, or vascular channels, may also be present, which are usually not seen in fibroadenomas [10]. Tumoral PASH often shows posterior acoustic enhancement due to its well-defined borders and less dense internal structure.

Phyllodes tumors, also known as cystosarcoma phyllodes, are rare fibroepithelial tumors primarily found in breast tissue but can occur in other soft tissues as well. While phyllodes tumors are usually rapidly progressive, in this instance, the lump has remained the same size for the past four years, indicating a slow progressive nature. These tumors are characterized by their leaf-like architecture and are classified as biphasic tumors due to the presence of both stromal and epithelial components.

Histologically, PASH is characterized by a distinct pattern of myofibroblast proliferation with slit-like spaces, which is crucial for diagnosis [11]. Immunohistochemical profiling is essential for differentiating PASH from low-grade angiosarcoma, with specific markers such as CD34, vimentin, desmin, actin, and progesterone receptor aiding in the correct diagnosis and preventing misinterpretation. Excision biopsy is the preferred method for obtaining adequate tissue for histopathological analysis, as fine-needle aspiration cytology (FNAC) and even core needle biopsies may not provide sufficient information.

A retrospective study published in 2013 concluded that PASH primarily affects premenopausal and perimenopausal women suggesting a hormonal basis for its development. For larger masses, conservative surgery was recommended alongside careful observation for patients at low risk for breast cancer [12].

An article published in 2020 showed an increased prevalence of PASH in up to 50% of men with gynecomastia suggesting a hormonal origin. Additionally, the interaction between medications like clonazepam, valproate, and risperidone with progesterone may elevate progesterone levels, potentially promoting the growth of PASH [13].

A similar case report demonstrates the atypical presentation of PASH, a rare, benign mesenchymal proliferation with a mass. An operative approach of excision or enucleation was preferred taking into account the possibility of upstaging to malignancy [14].

In this case of PASH concurrent with phyllodes tumor, management typically involved excision with clear margins. The presence of PASH does not change the fundamental treatment approach but underscores the importance of a thorough histological examination to exclude other conditions and ensure complete tumor removal.

## Conclusions

The coexistence of PASH within a phyllodes tumor underscores the importance of comprehensive histopathological evaluation in breast tumors. While PASH is a benign entity, its association with phyllodes tumors requires careful consideration to ensure accurate diagnosis and appropriate management. This case highlights the complexity of breast stromal lesions and emphasizes the need for pathologists to remain vigilant for unusual findings that may influence clinical outcomes. The biphasic nature, rapid growth, and potential for significant local and distant spread of phyllodes tumor necessitate a tailored approach to diagnosis and treatment. But this case is remarkable as phyllodes presented as a slowly progressive benign tumor. The rarity of these tumors underscores the importance of multidisciplinary care, including surgical, oncological, and pathological expertise, to ensure effective treatment and follow-up strategies. Continued research into the molecular mechanisms underlying phyllodes tumors and potential targeted therapies may further enhance management strategies and improve prognostic outcomes for affected individuals.
